# Developing prognostic models that predict onset and progression rates in genetic neurodegenerative diseases: perspectives of healthcare professionals in genetic counselling

**DOI:** 10.1186/s13023-026-04396-1

**Published:** 2026-05-13

**Authors:** Max J. Rensink, L. L. E. Bolt, A. Tibben, M. Oosterloo, B. P. C. van de Warrenburg, M. H. N. Schermer

**Affiliations:** 1 Department of Public Health – Ethics, Philosophy and History of Medicine Group, Erasmus Medical Centre, Dr. Molewaterplein 40, Rotterdam, 3015 GD The Netherlands; 2 Department of Clinical Genetics, Leiden University Medical Centre, Leiden, The Netherlands; 3 Department of Neurology, Maastricht University Medical Centre+, Maastricht, The Netherlands; 4 Institute for Mental Health and Neuroscience, Maastricht University, Maastricht, The Netherlands; 5 Department of Neurology, Donders Institute for Brain, Cognition and Behavior, Donders Center for Medical Neuroscience, Radboud University Medical Center, Nijmegen, The Netherlands; 6 Erasmus School of Health Policy and Management, Erasmus University, Rotterdam, The Netherlands

**Keywords:** Prognostic model, Age of onset, Disease progression, Hereditary neurodegenerative disease, Medical ethics, Predictive genetic testing

## Abstract

**Background:**

Research on the development of prognostic biomarker-based models for genetic neurodegenerative diseases lacking disease-modifying treatments is advancing rapidly. Currently, prognostic models are being developed to predict individual disease onset and progression rates for Huntington’s disease, spinocerebellar ataxia type 1, and spinocerebellar ataxia type 3. These models have potential applications in research, such as evaluating onset-delaying treatments and their timing, and in clinical practice, where they may offer personal utility to mutation carriers. However, the perspectives of healthcare professionals in genetic counselling have not yet been adequately explored.

**Methods:**

In this qualitative interview study, we interviewed 18 Dutch healthcare professionals in genetic counselling, representing the majority of the Dutch counselling practice for these conditions, to explore their views on using onset and progression predictions in clinical practice.

**Results:**

Healthcare professionals highlighted potential benefits, such as reducing uncertainty and supporting informed life and family planning, which are among the pivotal reasons for mutation carriers to opt for predictive genetic testing. They also noted possible drawbacks, including psychological distress for mutation carriers, discussed challenges in defining clinical disease onset, and some questioned the degree of personal utility of these predictions. Finally, healthcare professionals emphasised several key conditions for implementing such prognostic models, including sufficient predictive accuracy, a narrower and more precise age range, and the need for additional counselling.

**Conclusion:**

This study provides insights in the perspectives, views, and concerns of the majority of healthcare professionals in the Dutch counselling practice regarding the use of onset and progression predictions. Healthcare professionals identified several potential benefits and drawbacks of onset and progression predictions and discussed the conditions necessary for their implementation. During model development and before clinical implementation, clarity is needed on how disease onset is defined in the model and what constitutes sufficient model performance. Furthermore, aligning the model with its intended goals, such as providing personal utility, and assessing the feasibility of achieving meaningful predictions are important steps.

**Supplementary Information:**

The online version contains supplementary material available at 10.1186/s13023-026-04396-1.

## Background

Huntington’s disease (HD) and spinocerebellar ataxias (SCAs) are rare, autosomal dominant neurodegenerative diseases that typically manifest in adulthood and currently have no cure [1, 2]. In HD and some SCAs (e.g., SCA1 and SCA3), an expanded CAG repeat underlies disease development. Longer CAG repeat lengths correlate inversely with the age of onset of motor symptoms [3], but onset varies widely on an individual level. Prediction models based on CAG repeat length, such as the CAG-Age Product (CAP) score for HD [4, 5] and the Severity factor (S-factor) and multimodal models for SCAs [6, 7], are being used in research settings, for example for selecting participants. Neuroimaging has also been studied as a tool for onset and progression prediction in research contexts and clinical trial design [8, 9]. Although these prediction tools are valuable in research settings, they have not yet achieved sufficient performance for individual onset and progression prediction in clinical practice [10, 11].

HD and SCAs often exhibit full penetrance^1^, meaning that individuals carrying an expanded CAG repeat are certain to develop disease symptoms in the future. In HD, symptoms typically emerge between ages 30 and 50, leading to death within approximately 15 to 20 years after clinical diagnosis [1]. Main clinical manifestations include motor dysfunction like chorea, psychiatric disturbances such as depression and personality changes, and progressive cognitive decline [1]. In SCAs, symptoms vary by subtype, but central characteristic features are coordination disturbances and imbalance, which form the basis of clinical diagnosis. Additionally, speech difficulties and, in some cases, progressive cognitive impairment may occur [2]. At-risk individuals of HD and SCAs, such as those with a (grand)parent carrying the mutation, have the option to undergo predictive genetic testing (PGT) to determine whether they carry an expanded CAG repeat. While no cure exists, PGT can provide personal utility^2^. Primary motivations for PGT are reducing uncertainty and informing life and family planning [14, 15].

Currently, the Dutch research consortium CureQ (http://www.CureQ.nl) aims to develop prognostic models beyond CAG repeat expansion size to better predict onset and disease progression rates in HD, SCA1, and SCA3 [15]. These models can be useful in evaluating potential onset-delaying treatments in clinical trials and, if such a treatment becomes available, to determine the optimal timing for intervention. Since neurodegenerative processes in these diseases are believed to begin years or even decades before clinical onset [16, 17], early intervention in the presymptomatic or prodromal phase may be necessary. Beyond research and clinical benefits, prognostic models may also provide personal utility by offering carriers more detailed insights into their future disease trajectory.

The development of prognostic models that predict onset and progression rates marks a new phase in genetic testing. While PGT can inform at-risk individuals about whether they carry the mutation and may provide carriers with an approximate, group-level estimate of age of onset based on CAG repeat length, new models could make it possible to offer individualised predictions about the onset and progression of their future disease. It is therefore important to explore the views and perspectives of those directly involved. Over the past two decades, bioethics research has shifted from predominantly theoretical work to incorporating empirical insights, often referred to as the ‘empirical turn’ in bioethics [18, 19]. Stakeholder perspectives are increasingly recognised as essential for understanding the real-world impact of emerging technologies. Therefore, asymptomatic mutation carriers of HD, SCA1, and SCA3 have been interviewed to explore their perspectives on these prognostic models [20]. According to them, onset and progression predictions may inform life planning, reduce uncertainty and hypervigilance, help prepare for the disease, and inform family members. Reasons against receiving onset and progression predictions included concerns about potential negative psychological impacts, the expectation of similar disease progression rates as in family members, and a preference to receive progression information at a later stage.

We build on that study by investigating the views and perspectives of healthcare professionals (HCPs) involved in the presymptomatic genetic counselling for HD, SCA1, and SCA3. Literature on HCPs’ perspectives regarding clinical implementation of onset or disease progression predictions is limited to symptomatic patients, with studies focusing on physicians’ perspectives on personalised prognosis for patients with symptomatic amyotrophic lateral sclerosis (ALS) [21, 22], prognostic algorithms in advanced care planning for cancer patients [23], and personalised end-of-life prediction models in end-of-life care [24]. Gaining insight into perspectives of HCPs involved in presymptomatic genetic counselling is necessary for the ethically responsible implementation of prognostic models that predict onset and progression rates in clinical practice.

## Methods

This multicentre, qualitative interview study adhered to the COREQ checklist for qualitative research [25].

### Study design and recruitment

Dutch-speaking HCPs in the Netherlands who are involved in the presymptomatic genetic counselling for HD, SCA1, or SCA3 were recruited from June to November 2024. CureQ consortium members were excluded. To gain a wide range of perspectives, 18 HCPs with diverse professional backgrounds were enrolled. We aimed for recruiting HCPs who perform genetic counselling for these diseases, such as clinical geneticists, psychosocial workers, neurologists, specialised nurses, and psychologists. Where possible, interviews took place at the HCPs workplace, and otherwise online.

For recruitment, clinicians involved in CureQ were requested to approach potential participants directly or, with consent, to provide their contact details to the interviewer (MR). Each potential participant was contacted by the interviewer via email and received the participant information letter, which was also discussed prior to the interview. Additionally, the information letter was circulated among clinical geneticists. Lastly, referral sampling was applied, allowing initial participants to suggest other HCPs for study participation. One HCP was invited via email but did not respond. HCPs were enrolled and interviews were conducted until data saturation was achieved.

An initial interview guide was drafted and discussed among several authors (MR, LB, and MS). After incorporating revisions based on these discussions, the guide was discussed again (among MR, LB, MO, and BvdW) to finalise the questionnaire. The interview guide translated to English can be found in Supplementary material 1 and covered four main topics: (1) Professional background and role in presymptomatic genetic counselling, (2) Perspectives on predicting the age of onset of the disease, (3) Perspectives on predicting disease progression rates, and (4) Necessary conditions for implementing such prognostic models in clinical practice.

### Interviews, coding, and analysis

From August to November 2024, 18 semi-structured interviews were conducted (duration length 33 to 53 mins). The interviews were held at the participants’ workplaces or online (see Table 1). One author with experience in interviewing conducted all interviews (MR: male, MD, PhD candidate medical ethics). Each interview was audio-recorded, transcribed verbatim, and subsequently pseudonymised. Field notes were taken after every interview. After four interviews, two interviews were discussed among MR, LB, and MS to evaluate the interview guide and to identify preliminary themes. MR and LB, both experienced in conducting qualitative research, independently coded and compared all transcripts. Discrepancies were discussed until a consensus was reached. The initial coding framework based on the interview guide was iteratively refined based on emerging themes in the data. NVivo version 12 software facilitated thematic data analysis. Towards the last interviews, the emerging themes were discussed among MR, LB, AT, and MS in various combinations.

**Table 1:** Characteristics of the healthcare professionals

	Number of participants (*n* = 18)
Profession	
Clinical geneticist	7
Neurologist	4
Psychosocial worker	3
Psychologist	2
Specialised nurse	2
Sex	
Female	13
Male	5
Involved in counselling of HD and SCAs	
Solely HD	2
Solely SCAs	1
Both evenly distributed	4
Mainly HD, but also SCAs	10
Mainly SCAs, but also HD	1
Interview took place	
At workplace	11
Online	7

### Ethical approval and informed consent

This study was granted a waiver on March 4th 2024 by the institutional research review board of the Erasmus MC, confirming that this study is exempt from the Dutch Medical Research Involving Human Subjects Act (WMO) (MEC-2022-0827). All participants provided (digitally) signed informed consent.

## Results

### Study population

The large majority of HCPs had experience in counselling for both HD and SCAs, though most reported more extensive exposure to HD (see Table 1). Several participants noted that SCA1 is particularly rare, leading to fewer interactions with individuals at risk for this subtype. SCA3 was encountered more frequently. Almost all participants noted that mutation carriers receive information about their CAG repeat length, which offers some insight into age of onset at a group level.

Four main themes emerged from the interviews: (1) Potential benefits of more precise onset and progression predictions; (2) Potential drawbacks of more precise onset and progression predictions; (3) Conditions for clinical implementation; (4) Defining disease onset and progression rates. The presented quotes include the participant’s number, profession, and whether the participant works with HD, SCAs, or both.

### General observations

First, nearly all HCPs had given considerable thought to clinical use of onset and progression predictions prior to the interviews, and their responses were often nuanced. For instance, when discussing the potential impact of these predictions on presymptomatic mutation carriers, they carefully weighted advantages and drawbacks. Second, a broad range of perspectives emerged, with HCPs from diverse backgrounds and professional roles emphasising different aspects of the predictions. Third, the HCPs frequently discussed similar potential benefits and negative effects for both types of predictions (see Table 2). Consequently, in the following sections, onset and progression rates are addressed together, with distinctions highlighted where HCPs considered them relevant. Finally, many HCPs pointed out significant differences between HD and SCAs that could influence how individuals perceive and experience onset and progression predictions. These distinctions are also emphasised where relevant.

**Table 2:** Potential benefits and drawbacks of onset and progression predictions

Potential benefits	Potential drawbacks
Life planning Family planning Career and education planning End-of-life planning * Future care planning # Relationship and loved ones Activity planning	Negative psychological effects Confronting Losing hope, leading to fatalism Too much focus on onset Considering themselves ill earlier
Reducing uncertainty	Urge to know, but potentially overlooking consequences
Favourable predictions	Inaccurate predictions
Using predictions as a diagnostic tool *	Psychiatric & cognitive symptoms most invalidating *

* mainly for HD

# mainly for SCAs

### Potential benefits of onset and progression predictions

#### Reducing uncertainty

Many HCPs stressed that reducing uncertainty is a central benefit of onset and progression predictions. While current PGT clarifies carrier status, it also raises new questions, such as the exact timing of disease onset. More precise predictions could help address this uncertainty. Additionally, HCPs noted that such predictions may provide mutation carriers with a sense of control and greater clarity about their future.

 Participant 1, clinical geneticist, mainly HD

[Regarding PGT, ] a lot of people say, ‘‘Well, at least I’ll have certainty.’’ But we often point out that you’re actually exchanging one uncertainty, about carrying the mutation, for another: ‘‘When will the disease start?’’ For some people, that’s very difficult, while for others, it’s actually a kind of relief, like: ‘‘Well, maybe it won’t be so bad.’’ I can’t really foresee – I mean, the people who choose PGT are generally those who want to know where they stand. So I think the majority of that group would also want more clarity about the age of onset.

#### Life planning

Nearly all HCPs discussed that more precise predictions of onset and progression rates could support individuals in making major life decisions. First, individual predictions may inform family planning, as HCPs observed that carriers often consider the likelihood of developing disease symptoms during the early years of future children. Second, many HCPs noted the potential to inform career and educational choices, such as pursuing specific career paths or starting a business. Third, primarily in HD, some HCPs highlighted the benefits of these predictions for end-of-life planning. These predictions can facilitate timely discussions on this matter with a HCP such as a general practitioner, especially since anosognosia can hinder decision-making. However, despite early discussions, several HCPs stated that successfully arranging euthanasia^3^ in HD would still be difficult in practice due to factors such as cognitive impairment, anosognosia, and shifting perceptions of symptom burden. Fourth, multiple HCPs highlighted that, especially in SCAs, onset and progression predictions can aid future care planning, such as when to seek professional support (e.g., physiotherapy, psychological support) or plan ahead for access to services, considering potential waiting lists. The predictions can also guide discussions with loved ones about involving doctors timely, choosing an appropriate living environment, or transitioning to a nursing home. Moreover, a few HCPs noted the potential impact of this information on relationship decisions and emphasised the potential value of onset and progression predictions for loved ones and (future) caregivers in reducing uncertainty and preparing for the future. Lastly, some also suggested that onset and progression predictions could assist in planning ‘bucket list’ activities, such as world travel.

 Participant 15, psychosocial worker, mainly HD

It [onset and progression predictions] could have serious impact. On family planning: would you want a second child? On career choices: what will you do with your career? Will you pursue further education or not? But also on romantic relationships: how do you deal with this together with your partner?

#### ‘Favourable’ predictions: ‘‘The silver lining of bad news’’

Some HCPs suggested that ‘favourable’ onset or progression predictions could bring relief and hope to presymptomatic mutation carriers. For those living in constant fear of developing the disease, a favourable prediction, such as a late onset, could provide much-needed breathing room.

 Participant 3, psychosocial worker, mainly HD

For Huntington’s, if someone tests positive in their late 20s, there’s sometimes an immediate fear: ‘‘I’m getting Huntington’s, and it could happen tomorrow or next week.’’ But that’s definitely not certain with a repeat length of 40 or 41. In fact, it’s not very likely that symptoms will occur around age 30. So, people live with anxiety from that point onwards. But if you could say: ‘‘It starts at age 55 in your case’’, I think that would provide some breathing room and help manage the anxiety of the moment.

#### Onset predictions may help diagnose disease: ‘‘Is this the start of the disease?’’

A few HCPs noted that onset predictions, particularly for HD, could assist in diagnosing the disease, as determining the exact clinical onset remains challenging (see also Defining disease onset and progression rates). This is especially challenging when carriers or at-risk individuals present with non-specific symptoms, such as agitation, poor sleep, or increased stress. Some HCPs suggested that a predicted onset in the very near future could help confirm that these non-specific symptoms are prodromal, offering reassurance about their cause. Conversely, a prediction of a distant onset could provide some peace of mind and encourage consideration of alternative causes.

 Participant 4, clinical geneticist, mainly HD

If you have obvious chorea, it’s clear. But for some of the symptoms, like having a short temper when you’re 35 years old with three young kids and no sleep… Well, you know, is that the beginning of HD or not? … I think it would help if you could offer some reassurance, or confirmation, like: ‘‘Yes, you’re right. What you’re feeling is not just because you’re stressed, but also because the disease has started.’’.

### Potential drawbacks of onset and progression predictions

#### Negative psychological effects: ‘‘A double hammer blow’’

Nearly all HCPs discussed negative psychological effects as potential negative consequences of onset and progression predictions. First, many HCPs emphasised that the predictions can be (intensely) confronting. Some HCPs noted that anxiety about the future is often prevalent, especially for HD carriers and their loved ones due to the prominence of psychiatric and cognitive symptoms. HCPs suggested that this could make onset and progression predictions for HD mutation carriers even more challenging to process. One HCP described an adverse prediction, such as imminent onset, after first receiving a positive PGT result as a ‘‘double hammer blow’’.

 Participant 7, clinical geneticist, solely SCAs

If someone already receives bad news and then also hears that symptoms will begin in the short term, all hope is lost. So that’s another concern… It would feel like being hit with two devastating blows. We really need to think carefully about this. And when do we deliver such doubly bad news?

Second, as mentioned in the previous quote, several HCPs noted that burdensome predictions could cause individuals to lose hope. They emphasised that uncertainty about onset and disease progression is not necessarily problematic: it can offer hope to some carriers and at-risk individuals, allowing them to imagine a late onset or favourable progression rates. A few HCPs expressed concern that onset and progression predictions could therefore lead to fatalism, causing carriers to feel that their future is already determined.

 Participant 3, psychosocial worker, mainly HD

If you hear you fall into the unfavourable group – ‘‘Your condition will progress rapidly, starting with behavioural changes and cognitive decline’’ – then it seems fixed, you can’t influence it, and it’s coming your way. At that moment, you lose something again. You lose the hope of thinking: ‘‘Maybe it is a slow process, maybe it starts physically.’’ When you hear, ‘‘No, that is not the case for you. For you, it will progress quickly, especially the cognitive decline.’’ You have to deal with that too. Where do you find hope in such a situation?

Third, some HCPs feared that presymptomatic mutation carriers would focus too much on the predicted onset. They emphasised that the disease’s start does not represent ‘the end’ and that it is still possible to engage in meaningful activities. Some also pointed at other life events, such as an unrelated other disease that becomes so severe that it leads to death before ever experiencing symptoms of the neurodegenerative disease.

 Participant 6, neurologist, mainly SCAs

Because you focus on one thing: ‘‘Thén it will happen.’’ Life becomes entirely centred around that one thing, but then something completely different occurs. … Of course, it is a part of life, but you should allow it to remain just one part and not let it become the whole.

Lastly, a few HCPs expressed concern that onset and progression predictions could cause mutation carriers to perceive themselves as ill earlier. This mindset might discourage them from doing things they enjoy, and subsequently lead them to adopt a ‘sick role’. In addition, they stated that as carriers approach the predicted onset, they might become hypervigilant and start attributing seemingly normal events, such as a moment of forgetfulness or disbalance, to the disease.

 Participant 5, neurologist, mainly HD

There are also people who completely identify with the role of being a patient. If you can fairly accurately predict the onset, I think there are people who will cling to that completely. And they don’t give themselves the space to distance themselves from the disease while they still can.

#### The urge to know: ‘‘Will carriers oversee all consequences?’’

Some HCPs questioned whether individuals would fully grasp the potential consequences of onset and progression predictions, including the negative psychological effects discussed in the previous section. Several HCPs noted that once carriers choose to undergo PGT, it might be difficult for them to decline more precise information about their future if it is available. Some HCPs expressed doubt about whether all carriers are able to determine whether this information will truly benefit them.

#### Inaccurate predictions: ‘‘It might offer illusory certainty’’

Many HCPs stated that a downside of onset and progression predictions is their potential inaccuracy, which could undermine many of the possible benefits. Since one goal of these predictions is to provide carriers with personal utility, such as informed life planning, inaccuracies could lead to poorly informed choices. Additionally, some HCPs expressed concern that some carriers might struggle to fully grasp the uncertainty associated with the predictions (see also Necessary conditions for clinical implementation of onset and progression predictions). They might expect to gain complete certainty from these predictions, whereas some uncertainty, such as an age range or a probability, will always remain.

 Participant 20, neurologist, solely HD

I think that’s certainly a major concern in general when considering whether this is something you should want. That you may predict this for people. Because it will never be 100% accurate, some individuals will always be exceptions, and I believe that can have a significant impact.

#### ‘‘In HD, psychiatric and cognitive symptoms are considered more burdensome’’

Several HCPs pointed out that HD mutation carriers often consider the psychiatric and cognitive symptoms more burdensome than motor symptoms. However, the diagnostic criteria for disease onset following from the Unified Huntington’s Disease Rating Scale (UHDRS) and Scale of Assessment and Rating of Ataxia (SARA) focus primarily on motor symptoms and coordination disturbances. Although it is yet unknown how the prognostic models being developed will define disease onset, a few HCPs questioned the value of only predicting motor onset in HD.

 Participant 12, psychologist, solely HD

If I had to tell someone, ‘‘By the time you are forty, you can expect to become less mobile, or experience involuntary movements,’’ and that person has already personality changes, cognitive decline, depression, or irritability since the age of thirty-two… What would it really matter to someone if, by the age of forty, they also experience some motor symptoms on top of that?

### Necessary conditions for clinical implementation of onset and progression predictions

#### High accuracy: ‘‘There will always be some level of inaccuracy’’

Almost all HCPs stressed the importance of high accuracy. They used a variety of terms related to a model’s accuracy, including sensitivity, specificity, positive predictive value, negative predictive value, and confidence intervals. Nearly all agreed that the accuracy of the predictions should exceed 90%, with many suggesting over 95%, and some even advocating for 98%. However, there was scepticism about whether such levels of accuracy are truly obtainable, and some HCPs explicitly stated that achieving 100% accuracy is impossible. A few HCPs discussed the model’s accuracy and age range together, referring to 95% confidence intervals. In their view, higher accuracy corresponds to a narrower age range:

 Participant 5, neurologist, mainly HD

Oh, that’s very difficult to say [what the accuracy should be]. Every form of inaccuracy – because that’s what we’re talking about – comes with its own issues; I can’t say that the confidence interval needs to be x years. At the moment [on the basis of CAG repeats only], you’re essentially talking about confidence intervals of 20 years up or down, right? If it were 5 years, that would already be very good. It’s hard to say. There will always be some level of inaccuracy and unreliability, and within that uncertainty, people have to make their choices.

#### More precise age range

Many participants emphasised the importance of a more precise age range regarding onset predictions compared to the current onset estimates based on CAG repeat length. Most HCPs indicated that an ideal margin would fall between 4 and 6 years, for example an onset prediction between the ages of 37 and 42. A few HCPs considered a margin of up to 10 years as acceptable, while one participant suggested it should be narrowed to 2 to 3 years. Another participant noted that the preferred range might vary between individuals.

#### Additional counselling: ‘‘Some new topics need to be addressed’’

Multiple HCPs emphasised the need for additional counselling and highlighted several questions that need to be addressed before clinical implementation of onset and progression predictions (see Table 3). First, discussion should take place on who will provide the counselling and how it will be delivered. For example, some HCPs suggested that presenting onset and progression predictions together can offer a more comprehensive understanding of the future disease trajectory. Others, however, believed these predictions should be provided separately, as some individuals may prefer to receive only one of the two. Second, HCPs noted that careful consideration is needed regarding the timing of discussing to undergo onset and progression predictions (e.g., before or after receiving a PGT result), and when to disclose prediction results: alongside the PGT result or at a later time? Finally, multiple HCPs stressed the importance of carefully considering how to communicate these predictions.

**Table 3:** Conditions for clinical implementation of onset and progression predictions

**High accuracy:** ≥ 90%
**Precise age range**: 4–6 years
**Additional counselling needed** - *Who should provide counselling regarding onset and progression predictions*,* and should these predictions be combined or offered separately?*
- *When is the appropriate time to discuss the option of receiving onset and progression predictions?*
- *When is the appropriate time to discuss onset and progression prediction results?*
- *How can onset and progression prediction results be communicated effectively?*
**Broadly applicable**

 Participant 14, clinical geneticist, mainly HD

I still think that, as counsellors, we must carefully reflect on this [how to communicate onset and progression predictions] and consider the best approach, as there are many different ways to convey this information. It makes a difference whether you tell someone aged 25, ‘‘[the disease starts] between ages 35 and 40’’ or ‘‘in 10 to 15 years’’. That’s a completely different way of presenting it.

#### Other conditions

One HCP stressed the importance of ensuring that onset and progression prognostic models are applicable to and accurate for all patients, rather than being limited to specific groups, such as those defined by ethnic background. Another HCP raised concern about the possibility of unsolicited findings, particularly when using genetic biomarkers, and suggested that discussion should take place on how to handle such findings appropriately^4^.

### Defining disease onset and progression rates

Nearly all HCPs discussed the challenge of establishing the clinical onset of these neurodegenerative diseases in a timely manner, emphasising that their insidious nature makes it difficult to pinpoint the start of the disease. Some HCPs emphasised that this issue extends to the UHDRS and SARA assessment scales.

 Participant 6, neurologist, mainly SCAs

I find this a challenging concept [using the SARA for defining someone as symptomatic] because, prior to reaching the threshold, individuals labelled as presymptomatic may already exhibit symptoms such as spasticity, cramps, or polyneuropathy. These signs can precede other symptoms of the condition, like ataxia. I would consider those individuals symptomatic. However, we have collectively agreed that SCA patients are only considered symptomatic when we can objectively measure ataxia symptoms. So, I see this as a point of debate.

Several participants emphasised that cognitive and psychiatric symptoms in HD are often perceived as more burdensome than motor symptoms (see also In HD, psychiatric and cognitive symptoms are considered more burdensome), although the clinical diagnosis predominantly focuses on motor symptoms. Some HCPs therefore wondered which ‘onset’ the prognostic models being developed will predict.

 Participant 14, clinical geneticist, mainly HD

My question to you is: ‘‘Okay, but what exactly are you going to predict?’’ Can you also include that initial phase, which may actually have a greater impact than the movement symptoms, or rather, definitely has more impact than all those movement symptoms? What can you guarantee with your predicted age of onset? Which onset will we disclose to people: the movement disorder, or everything that precedes it?

Regarding disease progression rates, some HCPs stated that functional impairment or ‘disease milestones’ like falls, onset of dementia, loss of independence, or nursing home admission are the most relevant concern for mutation carriers. However, functional impairment is only partly assessed by the rating scales, which makes it highly challenging to predict. For instance, one HCP explained that there is often little correlation between the findings from neurological examination and a patient’s actual capabilities.

 Participant 5, neurologist, mainly HD

On the one hand, you have people with back pain who can do nothing and are fully disabled. But once I saw a someone with an inherited spastic paraparesis, and incredibly stiff legs, who could barely shuffle down the hallway. Yet, the person was still driving a truck and carrying heavy pallets. … What we objectively see in these individuals aligns very minimally with what they themselves feel they can do or would like to do. There is a discrepancy: while we use rational biological prediction models to describe biological and clinical phenomena, the relationship with function and how people organise their lives remains relatively weak.

## Discussion

To the best of our knowledge, this is the first study to explore HCPs’ perspectives on onset and progression predictions for presymptomatic mutation carriers of autosomal dominant adult-onset neurodegenerative diseases lacking a disease-modifying treatment. Multiple prognostic models have been developed for various neurodegenerative diseases, such as Parkinson’s disease [29] and ALS [30]. However, these models primarily focus on predicting disease risk or disease progression in symptomatic individuals, and studies into the insights of HCPs on implementing onset and progression prediction models for neurodegenerative diseases in clinical practice seem to be lacking. Our study addresses this gap by examining HCPs’ views on onset and progression prediction for presymptomatic mutation carriers of HD, SCA1, and SCA3 in a clinical setting.

In our study, all HCPs highlighted several positive effects of using onset and progression predictions in clinical practice, including reducing uncertainty and informed life planning^5^. In the PGT literature on HD, reducing uncertainty and life and family planning are among the pivotal reasons for testing [31, 32]. Similarly, our previous study involving asymptomatic mutation carriers of HD, SCA1, and SCA3 found that these aspects may be important motivations for seeking more precise information on disease onset and progression rates [20].

Several HCPs discussed as a potential benefit that onset and progression predictions could be used as a diagnostic tool, particularly when carriers or at-risk individuals present with subtle, non-specific symptoms. However, the primary goal of offering onset and progression predictions in clinical practice in the absence of a disease-modifying treatment is to offer personal utility to carriers. By using a *prognostic* model as a *diagnostic* tool, there is a risk of ‘function creep’^6^, whereby the purpose of the model shifts or expands [33]. Prognostic and diagnostic models differ in their design, objectives, and outcomes. A prognostic model aims to predict an outcome in the future and therefore relies on longitudinal data, whereas a diagnostic model is designed to identify current disease states, often using cross-sectional data [34, 35]. Given these differences, using a prognostic model for diagnostic purposes may lead to misdiagnoses or other harms, and careful deliberation is necessary before extending a model’s application beyond its original design.

HCPs discussed defining the moment of clinical onset as an important issue for both HD and SCAs. This raises the question of what onset and progression models can and should predict as ‘onset’. In the HD literature, there is ongoing debate about the role of nonmotor symptoms in confirming clinical diagnosis. Some researchers advocate for incorporating nonmotor symptoms as primary diagnostic criteria [10, 36], allowing for diagnosis in the absence of (unequivocal) motor signs. Others, such as McAllister et al. [37], oppose using nonmotor symptoms in clinical diagnosis, even though their study findings indicated that a significant portion of HD mutation carriers exhibits at least one cognitive or psychiatric symptom before and at motor onset (42.4% before and 64.8% at motor onset). Their concern centres on the challenge of distinguishing between psychiatric symptoms caused by HD and those arising from primary psychiatric disorders. This highlights the need for a discussion on what onset and progression models can and will predict, and to more fundamental and clinical-observational research in this regard^7^.

The HCPs highlighted three important concerns regarding the development of models that predict disease onset and progression rates: accuracy, personal utility, and feasibility. First, HCPs agreed that prognostic models, and thus the biomarkers they rely on, should achieve high levels of accuracy. However, some HCPs questioned whether this is possible, emphasising that prediction errors are inevitable. Similar concerns regarding accuracy have been raised by clinicians discussing the use of prognostic algorithms for cancer or end-of-life care [23, 24]. It raises the question of how much inaccuracy is acceptable: how many incorrect predictions, which, for example, could lead to misguided life planning, should be tolerated to provide personal utility for others? Furthermore, HCPs used different terms to describe the model’s accuracy, such as sensitivity, specificity, confidence intervals, and positive and negative predictive value. This indicates uncertainty about the specific model performance measures to be used and highlights the need for discussion on the model’s required performance for clinical implementation.

Second, for clinical implementation, it is necessary that the model’s predictions offer personal utility for mutation carriers. HD literature confirms the HCPs’ concerns that psychiatric and cognitive symptoms may be more invalidating than motor symptoms [38, 39]. If onset predictions would only predict motor symptoms and thereby reflect current clinical HD diagnosis [40], their meaningfulness for carriers may be limited. Additionally, some HCPs noted that predicting important disease milestones could also provide personal utility for carriers of HD and SCAs. However, the current assessment scales, which are probably most practical to use for model development given the large availability of data and their standardisation, do not sufficiently capture these aspects. For example, one-quarter of the current SARA measurements for assessing disease progression rates were considered not clinically meaningful by patients [41]. To conclude, it is necessary to consider how onset and progression predictions could offer the greatest personal utility for mutation carriers in clinical practice. This may differ from predictions that are meaningful in research settings, such as those aimed at evaluating potential onset-delaying treatments in clinical trials. The development of distinct models based on different goals may be required.

Third, it is important to assess the feasibility of models with most personal utility for carriers (e.g., predicting psychiatric and cognitive symptom onset in HD, or predicting disease milestones). Biomarkers indicating neuronal degeneration do not directly translate into specific clinical symptoms [42, 43]. Linking clinical symptoms to functional impairment presents further challenges, for instance because functional impairment is influenced by individual factors beyond the disease itself, such as occupation, daily activities, (psychological) resilience, and available care support [44, 45]. Furthermore, the available patient data on which these models will be trained may not be sufficient to provide such predictions. Once there is consensus on which predictions are most meaningful for mutation carriers, further research will be needed to assess their feasibility.

Potential negative psychological effects were highlighted by all HCPs and emerged also as a significant theme for asymptomatic mutation carriers in a previous study [20]. Some HCPs expressed concern that carriers might experience symptoms earlier after receiving an onset prediction^8^, potentially interpreting normal forgetfulness or tripping as a disease symptom. They feared that this could lead carriers to adopt a ‘sick role’, where they begin to behave as a patient, fulfilling the associated social expectations and interactions with family, friends, employers, and society at large [46]. In this way, onset prediction can have a nocebo effect and act as a self-fulfilling prophecy. Physicians discussing end-of-life prognosis models have expressed similar concerns [24], which have also been described in the literature on the experience of being at-risk for HD [47]. To mitigate this, additional monitoring and psychological support during follow-up care seems helpful. Moreover, HCPs stressed the importance of providing tailored counselling for onset and progression predictions and underscored the need for broader discussions regarding the organisation, timing, and content of such counselling. Existing guidelines for counselling in PGT do not address accurate, individualised onset and progression predictions [48]. Whether a staged disclosure process (for example, incorporating a standard waiting period between the PGT result and onset prediction result) would be beneficial or perceived as paternalistic is an open question. Nevertheless, extensive psychological support, as is provided in PGT, seems necessary for onset and progression predictions as well. To develop a framework for onset and progression predictions, communication guides such as the one proposed by van Eenennaam et al. [49] on personalised prognosis in ALS may serve as a starting point.

Our interviews revealed a somewhat paternalistic stance among HCPs, with some expressing concern that mutation carriers might urgently seek more precise prognostic information without fully weighing its consequences. Similar concerns have been raised in discussions on personalised prognosis for symptomatic ALS patients [21], where physicians raised concerns regarding the reliability of predictions but also worried about their potential to cause distress in patients and caregivers. However, distress following prognostic disclosure seems generally limited. While cases of severe regret following PGT have been reported in HD [50], a positive PGT result does not appear to be associated with long-term negative psychological effects^9^ [52]. Similar findings have been observed in other neurodegenerative diseases [53, 54]. Whether a more paternalistic approach is justified remains open for discussion.

A limitation of this study is that the field of genetic counselling for these diseases in the Netherlands is relatively small. We interviewed the majority of Dutch HCPs directly involved in this practice and included participants from diverse professional backgrounds, which allowed us to gather a wide range of perspectives. However, genetic counselling practices vary across countries, for instance due to differences in cultural norms and healthcare systems, which may have influenced our findings [55]. Future research may also explore the perspectives of HCPs internationally, especially when onset and progression prediction models for genetic neurodegenerative diseases are successfully developed. A further limitation of research into new technologies is that the prognostic models discussed are still in development, causing uncertainty about their final form. This can complicate the interviews, as it is unclear what the models will ultimately look like. However, the advantage is that it allows for the exploration of potential concerns, adjustments and/or improvements in an early stage.

## Conclusion

In this qualitative interview study, we explored the perspectives of Dutch HCPs in genetic counselling regarding the use of onset and progression predictions in clinical practice for autosomal dominant neurodegenerative diseases lacking a disease-modifying treatment. HCPs identified several potential benefits and drawbacks of onset and progression predictions and discussed the conditions necessary for their implementation. Overall, HCPs expressed some hesitation about implementing these models in clinical practice due to concerns about achieving sufficient accuracy, the difficulty in defining clinical onset, and the feasibility of providing personal utility to carriers. The insights gained from these interviews with HCPs in genetic counselling can contribute to the ethically responsible development and implementation of models predicting onset and progression in clinical practice.

## Electronic Supplementary Material

Below is the link to the electronic supplementary material.

Supplementary Material 1

## Data Availability

Data sharing is not applicable for this study due to privacy conditions.

## Footnotes

1 Some expanded CAG repeat lengths have a reduced penetrance. In that case, it remains uncertain whether an individual will develop disease symptoms in the future.

2 Multiple definitions of ‘personal utility’ exist [12]. The Institute of Medicine in the United States defined it as ‘the meaning and worth a genomic test brings to an individual from that individual’s perspective’ [13].

3 In the Netherlands, euthanasia performed by a physician is legally permitted under strict conditions [26]. Suffering from HD and SCAs can meet these criteria.

4 Frameworks for how to deal with unsolicited findings already exist, see for example Wouters et al. [27] and Stemkens et al. [28].

5 Although the HCPs in our current study did not explicitly discuss benefits for reduced penetrance carriers, onset and progression predictions could be particularly valuable for this group, as they still face uncertainty after PGT regarding whether they will develop disease symptoms in the future.

6 This can be defined as follows: ‘‘Function creep denotes an imperceptibly transformative and therewith contestable change in a data-processing system’s proper activity’’ [33].

7 Within the CureQ project, for example, onset and progression models will be partially trained on existing patient data, in which nonmotor features may not have been systematically captured.

8 Consider a 48-year old mutation carrier that receives aprediction of onset between ages 50 and 55. While relating potential prodromal symptoms to the disease, this individual might reduce engaging in potentially difficult tasks, for example at work, and might increasingly feel as if the disease has started already.

9 However, the high drop-out rates in the studies warrant some caution, as those who discontinue participation may experience greater psychological burden [51].

## References

[1] Caron NS, Wright GEB, Hayden MR. Huntington disease. GeneReviews^®^[Internet]. 1998. Last update: 2026

[2] Bhandari JTP, Samanta D. Spinocerebellar Ataxia. StatPearls [Internet]. 2023. 32491748

[3] Gusella JF, MacDonald ME. Huntington’s disease: the case for genetic modifiers. Genome Med. 2009;1(8):80. 19725930 10.1186/gm80 PMC2768966

[4] Langbehn D, Hayden M, Paulsen J. CAG-repeat length and the age of onset in Huntington disease (HD): a review and validation study of statistical approaches. Am J Med Genet B Neuropsychiatr Genet. 2010;153B(2):397–408. 19548255 10.1002/ajmg.b.30992 PMC3048807

[5] Warner JH, Long JD, Mills JA, Langbehn DR, Ware J, Mohan A, Sampaio C. Standardizing the CAP Score in Huntington’s Disease by Predicting Age-at-Onset. J Huntington’s Disease. 2022;11(2):153–71. 35466943 10.3233/JHD-210475

[6] Selvadurai LP, Perlman SL, Wilmot GR, Subramony SH, Gomez CM, Ashizawa T, et al. The S-factor, a new measure of disease severity in spinocerebellar ataxia: findings and implications. Cerebellum. 2023;22(5):790–809. 35962273 10.1007/s12311-022-01424-1 PMC10363993

[7] Petit E, Coarelli G, Morgan D, Cunha P, Hurmic H, Faber J et al. Predictive models for ataxia progression and conversion in spinocerebellar ataxia type 1 and 3. Brain. 2025. 10.1093/brain/awaf408 PMC12998449 41150672

[8] Farag M, Knights H, Scahill RI, McColgan P, Estevez-Fraga C. Neuroimaging Techniques in Huntington’s Disease: A Critical Review. Mov Disorders Clin Pract. 2025;12(5):561–76. 10.1002/mdc3.70010 PMC12070181 39976324

[9] Nigri A, Sarro L, Mongelli A, Castaldo A, Porcu L, Pinardi C, et al. Spinocerebellar ataxia type 1: one-year longitudinal study to identify clinical and MRI measures of disease progression in patients and presymptomatic carriers. Cerebellum. 2022;21(1):133–44. 34106418 10.1007/s12311-021-01285-0

[10] Ross CA, Reilmann R, Cardoso F, McCusker EA, Testa CM, Stout JC, et al. Movement disorder society task force viewpoint: Huntington’s disease diagnostic categories. Mov disorders Clin Pract. 2019;6(7):541. 10.1002/mdc3.12808 PMC6749806 31538087

[11] Klockgether T, Grobe-Einsler M, Faber J. Biomarkers in Spinocerebellar Ataxias. Cerebellum. 2025;24(4):104. 40411538 10.1007/s12311-025-01856-5 PMC12103481

[12] Caron NS, Wright GEB, Hayden MR. Huntington disease. GeneReviews^®^[Internet]. 2020.

[13] Kohler JN, Turbitt E, Biesecker BB. Personal utility in genomic testing: a systematic literature review. Eur J Hum Genet. 2017;25(6):662–8. 28295040 10.1038/ejhg.2017.10 PMC5477355

[14] Feero WG, Wicklund C, Veenstra DL. The Economics of Genomic Medicine: Insights From the IOM Roundtable on Translating Genomic-Based Research for Health. JAMA. 2013;309(12):1235–6. 23532238 10.1001/jama.2013.113

[15] CureQ. CureQ: Predict, Delay & Cure polyglutamine(Q) caused neurodegeneration 2022 [cited 22-10-2025]. Available from: https://cureq.nl/cureq-en/.

[16] Scahill RI, Zeun P, Osborne-Crowley K, Johnson EB, Gregory S, Parker C, et al. Biological and clinical characteristics of gene carriers far from predicted onset in the Huntington’s disease Young Adult Study (HD-YAS): a cross-sectional analysis. Lancet Neurol. 2020;19(6):502–12. 32470422 10.1016/S1474-4422(20)30143-5 PMC7254065

[17] Maas RPPWM, van Gaalen J, van de Klockgether T. The preclinical stage of spinocerebellar ataxias. Neurology. 2015;85(1):96–103. 26062625 10.1212/WNL.0000000000001711

[18] Borry P, Schotsmans P, Dierickx K. The birth of the empirical turn in bioethics. Bioethics. 2005;19(1):49–71. 15812972 10.1111/j.1467-8519.2005.00424.x

[19] Wangmo T, Hauri S, Gennet E, Anane-Sarpong E, Provoost V, Elger BS. An update on the empirical turn in bioethics: analysis of empirical research in nine bioethics journals. BMC Med Ethics. 2018;19(1):6. 29415709 10.1186/s12910-018-0246-9 PMC5803920

[20] Rensink MJ, Schermer MHN, Tibben A, Bijlsma EK, de Bot ST, Kievit JA, Bolt LLE. Exploring mutation carriers’ preferences regarding onset and progression of disease predictions for adult-onset genetic neurodegenerative diseases: a qualitative interview study. Hum Genet. 2025:1–13. 10.1007/s00439-025-02750-0 PMC12170675 40418244

[21] Moglia C, Palumbo F, Botto R, Iazzolino B, Ticozzi N, Calvo A, et al. Prognostic communication in amyotrophic lateral sclerosis: findings from a Nationwide Italian survey. Neurol Sci. 2024:1–8. 10.1007/s10072-024-07702-6 39073531

[22] van Eenennaam RM, Koppenol LS, Kruithof WJ, Kruitwagen-van Reenen ET, Pieters S, van Es MA, et al. Discussing personalized prognosis empowers patients with amyotrophic lateral sclerosis to regain control over their future: a qualitative study. Brain Sci. 2021;11(12):1597. 34942899 10.3390/brainsci11121597 PMC8699408

[23] Parikh RB, Manz CR, Nelson MN, Evans CN, Regli SH, O’Connor N, et al. Clinician perspectives on machine learning prognostic algorithms in the routine care of patients with cancer: a qualitative study. Support Care Cancer. 2022;30(5):4363–72. 35094138 10.1007/s00520-021-06774-w PMC10232355

[24] Hallen SAM, Hootsmans NAM, Blaisdell L, Gutheil CM, Han PKJ. Physicians’ perceptions of the value of prognostic models: the benefits and risks of prognostic confidence. Health Expect. 2015;18(6):2266–77. 24816136 10.1111/hex.12196 PMC5810722

[25] Tong A, Sainsbury P, Craig J. Consolidated criteria for reporting qualitative research (COREQ): a 32-item checklist for interviews and focus groups. Int J Qual Health Care. 2007;19(6):349–57. 17872937 10.1093/intqhc/mzm042

[26] Government of the Netherlands. Is euthanasia allowed? Ministry of Health, Welfare and Sport; [cited 10-30-2024]. Available from: https://www.government.nl/topics/euthanasia/is-euthanasia-allowed.

[27] Wouters RHP, Cornelis C, Newson AJ, Bunnik EM, Bredenoord AL. Scanning the body, sequencing the genome: Dealing with unsolicited findings. Bioethics. 2017;31(9):648–56. 28975656 10.1111/bioe.12375

[28] Stemkens D, van der Bruggenwirth HT. Dutch national consensus-based guideline for the disclosure of incidental findings during clinical genetic diagnostic testing. 2020.

[29] Li Y, McDonald-Webb M, McLernon DJ, Counsell CE, Macleod AD. Systematic review of prognostic models in Parkinson’s disease. npj Parkinson’s Disease. 2025;11(1):266. 40883335 10.1038/s41531-025-01112-x PMC12397436

[30] Xu L, He B, Zhang Y, Chen L, Fan D, Zhan S, Wang S. Prognostic models for amyotrophic lateral sclerosis: a systematic review. J Neurol. 2021;268(9):3361–70. 33694050 10.1007/s00415-021-10508-7

[31] Baig SS, Strong M, Rosser E, Taverner NV, Glew R, Miedzybrodzka Z, et al. 22 Years of predictive testing for Huntington’s disease: the experience of the UK Huntington’s Prediction Consortium. Eur J Hum Genet. 2016;24(10):1396–402. 27165004 10.1038/ejhg.2016.36 PMC5027682

[32] Goizet C, Lesca G, Dürr A. Presymptomatic testing in Huntington’s disease and autosomal dominant cerebellar ataxias. Neurology. 2002;59(9):1330–6. 12427879 10.1212/01.wnl.0000032255.75650.c2

[33] Koops B-J. The concept of function creep. Law Innov Technol. 2021;13(1):29–56.

[34] Moons KGM, Altman DG, Reitsma JB, Ioannidis JPA, Macaskill P, Steyerberg EW, et al. Transparent Reporting of a multivariable prediction model for Individual Prognosis or Diagnosis (TRIPOD): explanation and elaboration. Ann Intern Med. 2015;162(1):W1–73. 25560730 10.7326/M14-0698

[35] Van Smeden M, Reitsma JB, Riley RD, Collins GS, Moons KGM. Clinical prediction models: diagnosis versus prognosis. J Clin Epidemiol. 2021;132:142–5. 33775387 10.1016/j.jclinepi.2021.01.009

[36] Considine CM, Eddy CM, Frank SA, Kostyk SK, Oosterloo M, Killoran A, et al. Improving the clinical diagnostic criteria for genetically confirmed adult-onset Huntington disease: considering nonmotor presentations. Neurology: Clin Pract. 2025;15(2):e200427. 10.1212/CPJ.0000000000200427 PMC11741291 39830677

[37] McAllister B, Gusella JF, Landwehrmeyer GB, Lee J-M, MacDonald ME, Orth M, et al. Timing and impact of psychiatric, cognitive, and motor abnormalities in Huntington disease. Neurology. 2021;96(19):e2395–406. 33766994 10.1212/WNL.0000000000011893 PMC8166441

[38] Paoli RA, Botturi A, Ciammola A, Silani V, Prunas C, Lucchiari C, et al. Neuropsychiatric burden in Huntington’s disease. Brain Sci. 2017;7(6):67. 28621715 10.3390/brainsci7060067 PMC5483640

[39] Glidden AM, Luebbe EA, Elson MJ, Goldenthal SB, Snyder CW, Zizzi CE, et al. Patient-reported impact of symptoms in Huntington disease: PRISM-HD. Neurology. 2020;94(19):e2045–53. 32193209 10.1212/WNL.0000000000008906

[40] Oosterloo M, de Greef BTA, Bijlsma EK, Durr A, Tabrizi SJ, Estevez-Fraga C, et al. Disease onset in Huntington’s disease: when is the conversion? Mov Disorders Clin Pract. 2021;8(3):352–60. 10.1002/mdc3.13148 PMC8015887 33816663

[41] van de Maas R. Exploring the clinical meaningfulness of the Scale for the Assessment and Rating of Ataxia: A comparison of patient and physician perspectives at the item level. Parkinsonism Relat Disord. 2021;91:37–41. 34479057 10.1016/j.parkreldis.2021.08.014

[42] Koníčková D, Menšíková K, Tučková L, Hényková E, Strnad M, Friedecký D, et al. Biomarkers of neurodegenerative diseases: biology, taxonomy, clinical relevance, and current research status. Biomedicines. 2022;10(7). 10.3390/biomedicines10071760 PMC9313182 35885064

[43] Hansson O. Biomarkers for neurodegenerative diseases. Nat Med. 2021;27(6):954–63. 34083813 10.1038/s41591-021-01382-x

[44] Ross CA, Pantelyat A, Kogan J, Brandt J. Determinants of functional disability in Huntington’s disease: role of cognitive and motor dysfunction. Mov Disord. 2014;29(11):1351–8. 25216368 10.1002/mds.26012 PMC4197404

[45] Verbrugge LM, Jette AM. The disablement process. Soc Sci Med. 1994;38(1):1–14. 8146699 10.1016/0277-9536(94)90294-1

[46] Monaghan LF, Bury M. The sick role. Key Concepts in Medical Sociology. Third London: SAGE Publications, Inc; 2022. pp. 167–73.

[47] Tillerås KH, Kjoelaas SH, Dramstad E, von der Feragen KB. Psychological reactions to predictive genetic testing for Huntington’s disease: A qualitative study. J Genet Couns. 2020;29(6):1093–105. 32162754 10.1002/jgc4.1245

[48] MacLeod R, Tibben A, Frontali M, Jones A, Martinez-Descales A, Roos RA, et al. Recommendations for the predictive genetic test in Huntington’s disease. Clin Genet. 2013;83(3):221–31. 22642570 10.1111/j.1399-0004.2012.01900.x

[49] van Eenennaam RM, Kruithof WJ, van Es MA, Kruitwagen-van Reenen ET, Westeneng HJ, Visser-Meily JMA, et al. Discussing personalized prognosis in amyotrophic lateral sclerosis: development of a communication guide. BMC Neurol. 2020;20(1):446. 33308184 10.1186/s12883-020-02004-8 PMC7734773

[50] Hagberg A, Bui TH, Winnberg E. More appreciation of life or regretting the test? Experiences of living as a mutation carrier of Huntington’s disease. J Genet Couns. 2011;20(1):70–9. 20878217 10.1007/s10897-010-9329-6

[51] Timman R, Roos R, Maat-Kievit A, Tibben A. Adverse effects of predictive testing for Huntington disease underestimated: long-term effects 7–10 years after the test. Health Psychol. 2004;23(2):189. 15008664 10.1037/0278-6133.23.2.189

[52] Crozier S, Robertson N, Dale M. The psychological impact of predictive genetic testing for Huntington′ s disease: a systematic review of the literature. J Genet Couns. 2015;24:29–39. 25236481 10.1007/s10897-014-9755-y

[53] Paulsen JS, Nance M, Kim J-I, Carlozzi NE, Panegyres PK, Erwin C, et al. A review of quality of life after predictive testing for and earlier identification of neurodegenerative diseases. Prog Neurobiol. 2013;110:2–28. 24036231 10.1016/j.pneurobio.2013.08.003 PMC3833259

[54] Bemelmans SA, Tromp K, Bunnik EM, Milne RJ, Badger S, Brayne C, et al. Psychological, behavioral and social effects of disclosing Alzheimer’s disease biomarkers to research participants: a systematic review. Alzheimers Res Ther. 2016;8(1):46. 27832826 10.1186/s13195-016-0212-z PMC5103503

[55] Ormond KE, Laurino MY, Barlow-Stewart K, Wessels TM, Macaulay S, Austin J, Middleton A, editors. Genetic counseling globally: Where are we now? American Journal of Medical Genetics Part C: Seminars in Medical Genetics. Wiley Online Library; 2018. 10.1002/ajmg.c.31607 PMC5947883 29575600

