# Supplementary material for: Developing prognostic models that predict onset and progression rates in genetic neurodegenerative diseases: perspectives of healthcare professionals in genetic counselling
Source: Orphanet J Rare Dis. 2026 May 13;21:208. doi: 10.1186/s13023-026-04396-1 (PMC13221774; doi:10.1186/s13023-026-04396-1)
Supplement: Supplementary file 1 — Supplementary Material 1 [file 13023_2026_4396_MOESM1_ESM.docx]

**Supplementary Material 2: semi-structured interview guide** (translated to English)

*The interviews were conducted in Dutch. Subquestions were asked only when they were deemed relevant and if the participant had not yet addressed the specific question.*

**Background**

- Job, age
- How do you encounter mutation carriers and at-risk individuals for HD / SCA1 / SCA3 in your work? Are they symptomatic or asymptomatic?
- Do you usually discuss disease onset? If yes, what do you say? When?
- Do you inform them about the CAG repeat? If yes, what do you explain?
- Do you usually discuss disease progression? If yes, what do you say? When?

**Value of predictive information about age of onset**

- How would you define Age of Onset in the context of HD / SCA1 / SCA3?

Imagine that it becomes possible to predict the age at which symptoms will be clinically confirmed by a neurologist.

- What value would offering predictive onset information have for you as a healthcare professional, in clinical practice?

A mutation carrier asks you for an onset prediction.

- What do you think the impact of providing this information would be?
  - What are the benefits?
  - What are the risks / drawbacks?
  - What could be the influence on psychological wellbeing, family relationships, life decisions?
  - What could be the consequences for work?
- Do you think there is a difference between HD and SCA regarding the impact of onset information? Please explain.
- What could predictive onset information mean for at-risk individuals who currently do not want to be tested presymptomatically? What could the impact be for them?
- If such a test is developed, should predictive onset information also be offered to at-risk individuals? If yes, how?
  - At the same time as predictive genetic testing? Why or why not?
  - If this becomes available, should current (asymptomatic) mutation carriers be actively informed about this predictive information? Why or why not?
- What conditions should a test that provides onset information meet to be responsibly used in clinical practice?

The test result could, for example, provide a range in years: ‘‘Symptoms will begin between ages 38 and 41.’’ Another option is: ‘‘Symptoms will begin within the next 5 years.’’ Or: ‘‘Symptoms will begin at age 40.’’

- What do you think of these examples? Do you prefer one, or do you see other options?
- How reliable should the test result be? If 100 people receive an onset prediction, how often should it be correct?

**Value of predictive information about progression of disease (course of symptoms, speed of decline)**

- How would you define Progression of Disease in the context of HD / SCA1 / SCA3?

Disease progression can be explained in different ways. For example, by disease milestones such as a loss of mobility (when will someone need a wheelchair?). Imagine we can predict this with a test: loss of mobility, loss of independence, admission to a nursing home.

- What value would information about disease milestones have for you as a healthcare professional, in clinical practice?
- What do you think the impact of providing this information would be?
  - What are the benefits?
  - What are the risks / drawbacks?
  - What could be the influence on psychological wellbeing, family relationships, life decisions?
  - What could be the consequences for work?

Disease progression can also be explained as the speed of decline, i.e., how quickly someone will deteriorate.

- What value would information about the speed of decline have for you as a healthcare professional, in clinical practice?
- What do you think the impact of providing this information would be?
  - What are the benefits?
  - What are the risks / drawbacks?
  - What could be the influence on psychological wellbeing, family relationships, life decisions?
  - What could be the consequences for work?
- Do you think there is a difference between HD and SCA regarding the impact of progression information? Please explain.
- What could predictive progression information mean for at-risk individuals who currently do not want to be tested presymptomatically? What could the impact be for them?
- If such a test is developed, should predictive progression information also be offered to at-risk individuals? If yes, how?
  - At the same time as predictive genetic testing? Why or why not?
  - If this becomes available, should current (asymptomatic) mutation carriers be actively informed about this predictive information? Why or why not?
- What conditions should a test that provides predictive progression information meet to be responsibly used in clinical practice?
  - How reliable should the test result be?
  - How precise should predictive progression information be to be useful? Why?

**Closing**

- How do you assess the need for onset and progression information among mutation carriers and at-risk individuals?
- Do you have any additions? Is there anything else you would like to share?
- Who else should we speak with about this topic? (colleagues)
- May we contact you again if further questions arise?
